# Editorial: Sustainable catalytic production of bio-based heteroatom-containing compounds Volume III

**DOI:** 10.3389/fchem.2023.1210932

**Published:** 2023-05-02

**Authors:** Hongguo Wu, Hu Li, Yaqiong Su, Song Yang

**Affiliations:** ^1^ National Key Laboratory of Green Pesticide, Key Laboratory of Green Pesticide and Agricultural Bioengineering, Ministry of Education, State-Local Joint Laboratory for Comprehensive Utilization of Biomass, Center for R&D of Fine Chemicals, College of Pharmacy, Guizhou University, Guiyang, Guizhou, China; ^2^ School of Chemistry, Xi’an Key Laboratory of Sustainable Energy Materials Chemistry, State Key Laboratory of Electrical Insulation and Power Equipment, Xi’an Jiaotong University, Xi’an, China

**Keywords:** green chemistry, biomass and biofuels, heterogeneous catalysis, waste valorization, biological activity

Heteroatom-containing compounds are core scaffolds in a variety of medicines, and important building blocks for the synthesis of fine chemicals (Li et al., 2016; Meng et al., 2022; Wang et al., 2023a). As a promising alternative to fossil fuels, biomass sources rich in oxygen species can be used as versatile feedstocks for producing both biofuels and task-specific molecules (Li et al., 2018; Wang et al., 2023b; Huang et al., 2023; Meng et al., 2023). For the sustainable conversion processes developed for biomass upgrading, the used catalytic pathways and strategies are key to regulating product distribution. Also, increasing attention has been paid to investigating the reaction mechanisms using advanced or *in situ* characterization techniques and theoretical calculations. Moreover, environmental and energy issues (e.g., the greenhouse effect and energy depletion) encountered in the conventional use of fossil resources have arisen more concerns in the exploration of renewable biomass and waste CO_2_ for the manufacture of carbon-based functional materials/substances employing well-tailored catalysts or catalytic systems.

This Research Topic is Volume III of a series, and here we present a Research Topic of original research and review articles (12 papers in total) with topics on green and sustainable chemistry, including catalytic production of biodiesel (Zhang et al., and Pan et al.), catalytic upgrading of biomass derivatives (Wang et al., Li et al., Zheng et al., and Huang et al.), selective hydrogenation of CO_2_ to CH_4_ (Xiang et al.), bioactive assessment of natural products (Qu et al.), and environmental issues involved in the biomass utilization processes (Liu et al., Yang et al., Yang et al., and Yang et al.).

A research paper by Wang et al. reports the preparation of Pt-WO_*x*_ catalysts supported on TiO_2_ with different crystal forms and WO_*x*_ loadings for catalytic performance in the hydrogenolysis of glycerol to 1,3-propanediol. The anatase-type TiO_2_-supported catalyst (Pt/W/A-Ti) with higher stability shows superior catalytic performance to the rutile-type TiO_2_ catalyst (Pt/W/R-Ti). Also, the catalytic mechanisms are investigated by *in situ* characterization techniques, manifesting that glycerol is first transformed into 3-hydroxypropanal over Pt/W/A-Ti, followed by succeeding conversion steps to give 1,3-propanediol. Xiang et al. detailedly study the formation process of methane (up to 100% selectivity) from catalytic hydrogenation of CO_2_ at 280°C over a Ni-based ETS-10 zeolite catalyst, which is prepared by *in situ* doping and impregnation. Intensive characterizations and measurements are conducted to get insights into the catalyst promotional mechanisms, with attention to the impact of different Ni incorporation methods on the catalyst stability. In addition, the antifungal activities of the *Allium mongolicum* Regel leaf (Qu et al.), and the health risks of heavy metals in soils and food crops (Liu et al.) are assessed, as well.

This Research Topic also features several Mini reviews with varying scopes (Zhang et al., Pan et al., Li et al., Zheng et al., Huang et al., Yang et al., Yang et al., and Yang et al.). Zhang et al. summarize recent advances in the development of magnetic catalytic materials for producing biodiesel, with a focus on the catalyst physicochemical properties, performance, and recyclability. The involved catalytic mechanisms and reaction conditions in biodiesel production are discussed, and attention is also paid to the limitations and challenges for future research. Viewing that ionic liquid-functionalized materials have the unique characteristics of both homogeneous and heterogeneous catalysts, they are recently considered as one of the promising alternatives to conventional homogeneous acid/base catalysts for biodiesel production. Accordingly, a review on the topic of developing supported acid/base ionic liquids as heterogeneous catalysts for producing biodiesel is presented (Pan et al.). The merits and demerits of various supports (e.g., mesoporous silica, porous polymers, carbonaceous materials, metal-organic frameworks, and ferromagnetic materials) in biodiesel production are collected, and their performance in immobilization of ionic liquids is compared, with emphasis on the developed methods effective for immobilizing ionic liquids onto solid supports to prepare the functional ionic liquids. The ionic liquid-based solid catalysts are also reported to be efficient for the pretreatment of lignocellulosic biomass, and the obtained cellulose and hemicellulose components can be further converted to 5-hydroxymethylfurfural in the presence of the tailored acidic ionic liquids (Li et al.).

Instead of chemocatalysis, Zheng et al. make a brief introduction to the classification, source and application of alginate lyases in the biocatalytic degradation of carbohydrates, which primarily concentrates on screening of strains, mining of genes, and analysis of degradation substrate and product structure. In addition to the comprehensive utilization of saccharides, Huang et al. introduce the development of a variety of anodes (e.g., Pt, Pb, Ir, Ni, and Co) for electro-oxidative degradation of lignin, with emphasis on the product distribution caused by different electrodes, as well as the involved reaction pathways and catalytic mechanisms. For the construction of ecological civilization and the goal of carbon peak and carbon neutralization, several reviews regarding carbon effect calculation and upgrading strategy (Yang et al.), improvement of rural soil properties and states by biomass carbon (Yang et al.), and rural resilience research from the perspective of the ecosystem (Yang et al.) are also showcased.

We wish this Research Topic enlightens more sustainable and eco-friendly conversion pathways, sheds light on reaction mechanisms, and develops novel catalytic strategies for producing biofuels and high-value chemicals. Enjoy its reading!
